# Contextual Congruency Effect in Natural Scene Categorization: Different Strategies in Humans and Monkeys (*Macaca mulatta*)

**DOI:** 10.1371/journal.pone.0133721

**Published:** 2015-07-24

**Authors:** Anne-Claire Collet, Denis Fize, Rufin VanRullen

**Affiliations:** 1 Université de Toulouse, UPS, Centre de Recherche Cerveau et Cognition, Toulouse, France; 2 CNRS, CerCo, Toulouse, France; CNR, ITALY

## Abstract

Rapid visual categorization is a crucial ability for survival of many animal species, including monkeys and humans. In real conditions, objects (either animate or inanimate) are never isolated but embedded in a complex background made of multiple elements. It has been shown in humans and monkeys that the contextual background can either enhance or impair object categorization, depending on context/object congruency (for example, an animal in a natural vs. man-made environment). Moreover, a scene is not only a collection of objects; it also has global physical features (i.e phase and amplitude of Fourier spatial frequencies) which help define its gist. In our experiment, we aimed to explore and compare the contribution of the amplitude spectrum of scenes in the context-object congruency effect in monkeys and humans. We designed a rapid visual categorization task, Animal versus Non-Animal, using as contexts both real scenes photographs and noisy backgrounds built from the amplitude spectrum of real scenes but with randomized phase spectrum. We showed that even if the contextual congruency effect was comparable in both species when the context was a real scene, it differed when the foreground object was surrounded by a noisy background: in monkeys we found a similar congruency effect in both conditions, but in humans the congruency effect was absent (or even reversed) when the context was a noisy background.

## Introduction

Visual categorization appears to be a crucial ability for survival of many, especially diurnal, animal species, since they have to quickly adapt their behaviour to many different situations (to hunt a prey or escape from predators, for instance). However, because categorization implies abstraction, it was initially explored mainly in humans. In 1964 Herrnstein & Loveland [1] demonstrated for the first time a categorization capability in animals (pigeons). Following the pioneering work of Herrnstein & Loveland, other neuroscientists and behaviourists addressed the question of conceptualization and categorization in animals. The first studies investigated the existence of categories in animals at the subordinate or basic level. Thus, in 1984 Schrier et al [2] tested the existence of three concepts in macaques: “human”, “monkey” and” letter A”. They found clear evidence of transfer of the categorization rule to new items. In 1988, D’Amato & VanSant [3] also tested the existence of the concept “human” in monkeys; they showed that, if the picture training set is large enough, it allows generalization and thus, good transfer to new stimuli. But at that basic level of categorization, items within a category also share a lot of visual similarities, and abstraction abilities may not be necessary for correct performance. In 1988, Roberts and Mazmanian [4] explored the existence of a more abstract concept, “animal”, using different levels of categories in pigeons, non-human primates and humans. They showed that, after training, monkeys and pigeons could discriminate pictures well at all category levels (subordinate level: find one species of birds—the common kingfisher- among other birds; basic: find birds among other animals; super-ordinate: animals versus objects). However, they found weak categorical transfer to new stimuli in both species. Nonetheless, in that study the stimulus training set was limited (36 to 40 pairs of stimuli), and may not have been sufficient for optimal category learning, encouraging instead an association learning rather than a conceptualization. Then Fabre-Thorpe in 1998 [5] showed that non-human primates could categorize pictures according to the presence or absence of animals using a much larger set of stimuli (340), with a similar level of performance as humans. Such evidence suggests that high level abstraction exists in primates, because different images of animals vary a lot in terms of physical features (body shape, size…).

Moreover, Sands et al in 1982 [6] showed that this ability could emerge even without specific training, using a comparison task in monkeys. The same observation was made by Astley and Wasserman (1992) in pigeons [7]: birds had to learn by heart to discriminate a set of positively reinforced pictures among others, and they tended to make more discrimination errors when these pictures were tested against others belonging to the same category.

Researchers then tried to assess the diagnostic features on which animals could rely to efficiently categorize pictures. First, animals have to infer the categorization rule by trial-and-error, and both pigeons [8,9] and monkeys [3] appeared to better pick up relevant features for positively reinforced stimuli than for negative ones. On the other hand, the same teams showed that colours are not crucial features for categorization. Finally, while humans and monkeys can process stimuli in a global way [10], it was long held that pigeons only process visual information in a fragmented way, based on local features [9, 11], as their performance in categorization tasks can be quite robust against stimulus scrambling. However, this point of view was challenged by Wasserman and colleagues [12, 13]: they demonstrated that pigeons were sensitive to the spatial organization of objects. Furthermore the same team later found evidence that pigeons exhibited rotational invariance in object recognition, if trained with different viewpoints of the same stimulus [14], just as monkeys do [15]. Those results suggest that pigeons also can process visual stimuli in a global way.

Although many teams have worked on categorization, the neural mechanisms underlying this ability remain unclear. Nevertheless Kriegeskorte et al in 2008 [16] compared activation patterns in inferior temporal cortex in both humans and monkeys during a passive fixation task of isolated items. Despite the use of two different techniques (fMRI in humans and single cell recording in monkeys), they observed strong similarities between humans and monkeys in the way items are grouped into categories in IT (items belonging to the same category elicited activation patterns close to each other).

In the real world, however, objects are rarely isolated but surrounded by a complex environment with multiple elements. In difficult situations our a priori knowledge of a context could help detect and recognize a target object. Contextual information can thus affect the efficiency of object recognition [17,18]). Behavioural studies in humans have explored the importance of context in facilitation and enhancement of visual processing and perceptual memory. They demonstrated that, by manipulating context, it is even possible to create false memories [19] or to predispose subjects to false recognition of objects [20].

In the studies cited above, visual exposure to contexts was long enough to allow memorization or precise exploration. But Joubert et al in 2007 [21] showed that human subjects are equally fast at categorizing a scene at a super-ordinate level (for instance man-made scenes versus natural scenes) as an object (e.g. animals versus objects). However, it takes longer to categorize scenes at the basic level, for example sea, mountain, indoor or urban scenes [22], suggesting a coarse to fine processing of such stimuli. The same super-ordinate level advantage was found in object categorization tasks: it is faster to recognize a bird as an animal than as a bird [23]. Moreover, Joubert et al (2008) [24] showed that the congruency effect between a scene and an object appears very early in an ultra-rapid categorization task where stimuli are flashed very briefly, which suggests a parallel processing of both context and object information. Davenport & Potter [25] also came to this conclusion, by comparing scene and object categorizations in humans, either with mixed or isolated stimuli. They found better performance when foreground object and background were congruent, whatever the task, i.e. whether subjects had to categorize only the foreground object, only the background, or both. Moreover, a congruency effect between context and object has also been observed in monkeys during a rapid visual categorization task [26]: task performance (categorization of the foreground object as an animal/non-animal, where only stimuli containing an animal were the targets of the task) was affected both in terms of accuracy and reaction times, and in the same way in humans and monkeys. These results suggest that the brain mechanisms underlying the congruency effect may be similar in the two species.

The above-mentioned studies explored the time course of categorization and contextual influences, but did not directly probe the visual features of the scene images that could support these cognitive abilities. Indeed, a scene is not only a collection of objects; it also has a spatial arrangement, and global physical features which can contribute to defining its gist. Algorithms can efficiently categorize scenes on the basis of such global statistics, especially the shape of the spatial envelop [27]. This envelope corresponds to the outlines of elements of a scene which define its three-dimensional layout (like walls, buildings, mountain slopes, landscape relief etc) as well as their relations. In addition, after learning the power spectra of many different images, other algorithms were able to detect animals in different contextual scenes significantly above chance [28]. This leads to the question of what type of physical information is relevant for the human and primate visual systems to access context category. Joubert et al. in 2009 [29] designed a context categorization task in which they first equalized the amplitude spectra of their stimuli, and then destroyed the phase spectrum of the previously equalized pictures. They demonstrated that although amplitude equalization slightly impairs categorization performance in terms of reaction times, the most important diagnostic criterion relies on phase information. Loschky et al [30] also found that unlocalized amplitude information was insufficient to categorize scenes at the basic level in humans. On the other hand, Guyader et al [31] found that processing of scene category was influenced by amplitude spectrum information. They used a priming paradigm in a scene categorization task, and showed that chimeric primes built from an amplitude spectrum of scenes of the same category as the target image could speed up the categorization. Therefore, the relative contributions of these two global statistical dimensions (i.e. phase and amplitude) to the categorical processing of an image remain unclear.

In our experiment we aimed to explore the contribution of the Fourier amplitude spectrum to context-object congruency effects in humans and monkeys. To our knowledge, all previous studies comparing visual categorization in humans and monkeys concluded that processing of objects and scenes were very similar in both species.

We designed a protocol where images of isolated objects or animals were pasted either on real scene photographs (man-made or natural) or on noisy backgrounds built from Fourier transforms of 100 real scenes averaged in amplitude (and phase randomized) with a varying proportion of naturalness. The expected congruency effect was at the super-ordinate category level (i.e. natural scenes are congruent with animals and man-made scenes with objects). If at least part of this effect relies on power spectrum information, then we should observe better performances for congruent stimuli, i.e. animals pasted on a noisy background with a high degree of naturalness or objects pasted on a background with a low degree of naturalness, than for incongruent ones.

## Material and Methods

### Subjects

#### Non-human primates

Two male rhesus monkeys (Rx and Dy, both 20 years old) performed a rapid visual go/no-go categorization task: Animal versus Non-Animal. Both monkeys were previously trained for similar tasks. They both took part in the experiments of Fize et al, 2011 [26] and in the study of Fabre-Thorpe, 1998 [5], where monkey Dy performed a food/non-food categorization task (target category: food) and monkey Rx an animal/non-animal task (target category: animal). They were born in captivity, raised with congeners but no other species of animals or non-human primates. However, even though they had never been exposed to the animal species from the task in real life, they had previously watched wildlife documentaries during the early stage of their training for the experiment of Fabre-Thorpe, 1998 [5]. This could have helped them to form the natural category “animal”.

All procedures conformed to French and European standards concerning the use of experimental animals; all protocols used in this study (visual stimulation, training and experimental protocols, reward system, animal care) were approved by the regional ethical committee for experimentation on animals (agreement ref. MP/05/05/01/05, C2EA-14 ethical committee of Marseille). The agreement was delivered for a larger project on visual context processing in monkeys; this study was part of the project.

During the experimental period, animals are weighed every day before the session. If a loss of body mass larger than 5% is detected, dietary restriction is controlled in order to stabilize the animal’s weight. If a loss of body mass of more than 10% is detected, the experiment is stopped until the animal has recovered its initial weight. A veterinarian examines each animal once a year, performing blood tests to check for SIV, hepatitis A, HSV, STLV and also performing faecal cultures. The laboratory includes an elected committee responsible for animal well-being. This committee ensures that all lab members working with animals abide by the procedures in accordance with French and European ethical standards.

Animals are housed in indoor enclosures (surface of 12m^2^, height of 4 meters), which they share with 2 or 3 congeners of the same sex. The enclosures include perches of different heights and different adapted toys (balls, slides, swings). Each animal also has an individual cage (0.75m x 1m x 2m) that can be accessed via a tunnel from the enclosure. Monkeys are fed and watered in these cages where they spend about 6 hours every day. They are given dry macaque food (50g/kg of body weight), three fresh fruits and one vegetable. During the experimental period fruits are provided after the experimental session (regardless of performance during the session), water is also provided at the same time in an appropriate amount to complement what the animal drunk during the session. During weekends (when no experimental sessions are carried out) animals are on a normal diet including water, dry food, vegetables and fruits, provided at the same time as the other animals and in the same quantity as mentioned above. On weekends a single person is in charge of animal care, and monkeys spend only 3 hours in their cages.

Monkeys did not suffer from the experiment (since it was a psychophysical experiment, where neither surgery nor drug injection was required). Both monkeys were retired after this experiment but remained in the animal facility of the institute. One died a few months later from natural causes.

#### Humans

Fifteen human subjects performed the same go/no-go visual categorisation task with the same experimental device (7 men, mean age: 27 range 22–37, 2 of them left-handed). They all had normal or corrected to normal vision. All participants gave written informed consent prior to taking part in the experiment. The study was approved by the local ethics committee ‘‘CPP Sud-Ouest et Outre-Mer I” under protocol number 2009-A01087-50.

### Stimuli

#### Vignettes

We used objects (tools, furniture, vehicles) and animals (mammals, birds, reptiles, insects, fishes) vignettes (from Hemera Photo Objects library, tiff format). Monkey Rx saw 400 vignettes, Monkey Dy 320 and humans 260. For all subjects half of the vignettes were animals, whereas half were objects. The largest dimension of each vignette was scaled to 100 pixels (6.2° of visual angle), regardless of its identity.

#### Backgrounds

Vignettes were randomly pasted on a background: either a photograph of a real scene (either a man-made or a natural scene, 100 exemplars of each, Real scene condition) or on a noisy background with a varying level of naturalness (from 0% to 100% by steps of 10%, Noisy background condition).

To build these noisy backgrounds we averaged Fourier amplitude spectra of 100 real photographs, randomized the corresponding phase spectrum, and then reconstructed an image by an inverse Fourier transform. In order to create the 11 levels of naturalness, the proportion of natural and man-made scenes among the 100 photographs varied: for example, for level 20% we used 20 photographs of natural scenes and 80 of man-made scenes. Ten noisy backgrounds of each level were built from randomly chosen photographs. The photographs used to build noisy backgrounds were the same as those used in the Real scene condition.

**Fig 1:**
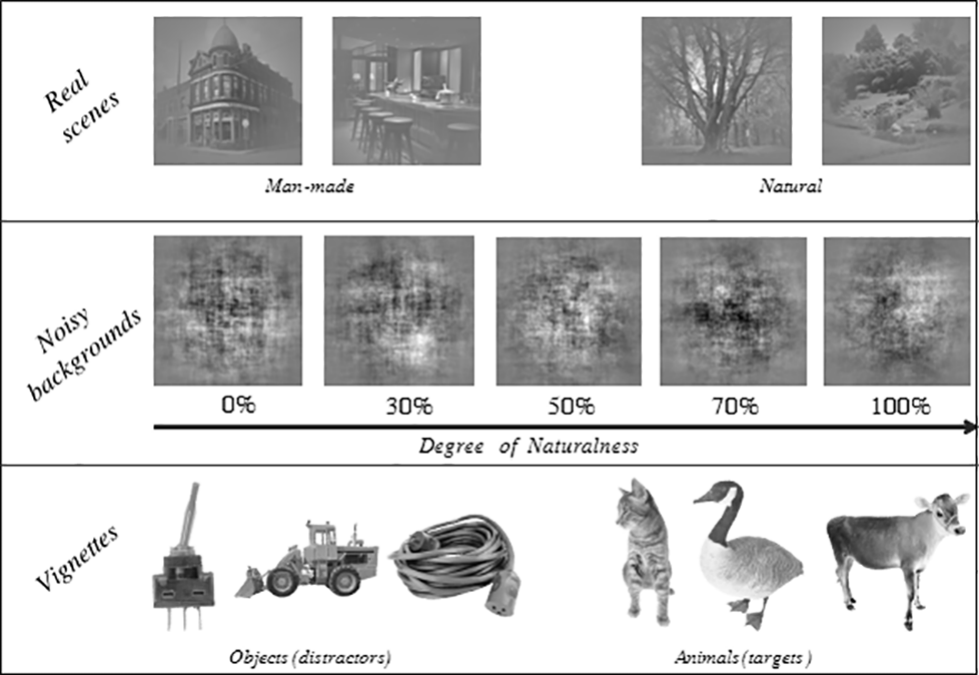
First line: examples of real scenes; second line: examples of noisy backgrounds with an increasing degree of naturalness; third line: examples of vignettes

Both backgrounds and vignettes were in greyscale (Fig 1), equalized in contrast and luminance. Stimuli were displayed on a grey screen (resolution 600x800 pixels). Backgrounds (size 600x600 pixels) had their borders smoothed using a Gaussian filter. Vignettes were pasted randomly in one out of nine possible positions (centre of the screen or on a circle, 3.2 degrees of eccentricity). By randomizing vignette locations and equalizing their sizes, we sacrificed the spatial and scale coherence of stimuli (e.g. a large mouse could be pasted in the middle of the sky, or a small elephant on a table top).

To maximise the influence of contextual effects, the transparency of vignettes was adjusted online based on subject’s performance using a stair-case function applied to the alpha layer. We fixed the threshold at 70% of accuracy on targets, regardless of trial condition (noisy or real backgrounds). Transparency values were confined between 0 (fully visible) and 0.6.

**Fig 2:**
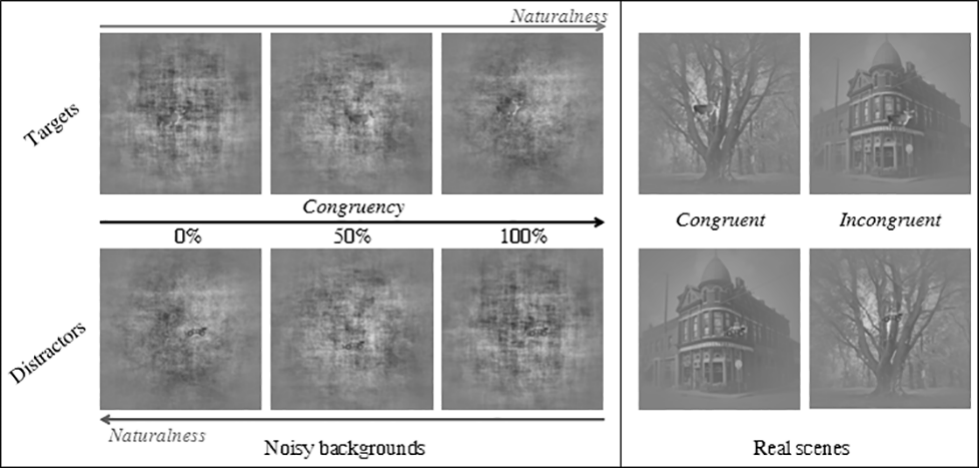
Examples of final stimuli used in the experiment (a cow as target, a tractor as distractor). The congruence level increases with the degree of naturalness for targets, but decreases for distractors. This figure also illustrates the size ratio between vignette and background, which makes the task difficult.

In the “Real scene” condition, we considered targets as congruent with a natural scene and distractors with a man-made scene. The two other associations were labelled as incongruent (Fig 2).

In the “Noisy background” condition, the 11 levels of naturalness allowed us to build 11 levels of congruency: for example, a noisy background of 20% naturalness corresponds to a 20% level of congruence for targets and an 80% level for distractors (Fig 2).

Subsequently, data were analysed according to the congruency level.

### Procedure

Subjects (both monkeys and humans) performed a rapid visual go/no-go categorization task. They sat in a dimly lit room 50cm away from a computer screen (resolution 600x800 pixels, vertical refresh of 60 Hz). Stimulus display and behavioural response measurement were carried out using the Psychtoolbox of Matlab 7.6.0 (R2008a).

Each trial started with a fixation point (4x4 pixels) displayed at the centre of a grey screen for 800ms, immediately followed by the stimulus for 3 frames (50ms). To start the experiment, subjects placed their fingers (preferred hand) on a response pad equipped with infrared electrodes that provided timing information with millisecond precision. For each stimulus containing a target (i.e. an animal), subjects had to release the pad within 800ms; longer reaction times were considered as no-go responses. If the stimulus did not contain an animal but an object, subjects had to keep their fingers on the pad, and so inhibit any motor response.

When subjects made a mistake, the corresponding stimulus reappeared for 1500ms (negative feedback). There was no positive feedback for humans, but monkeys were rewarded with a drop of juice following each correct response (either go or no-go).

Subjects could suspend the experiment whenever they wanted, by releasing the pad. All conditions were randomly mixed.

Monkeys worked every day ad libitum during 5 weeks (Monkey Rx performed 16800 trials, and monkey Dy 18750 trials); reward was gradually increased during a session to maintain the motivation level. In order to avoid the possibility of learning a specific set of stimuli, 10 new vignettes were added every day, while the set of backgrounds remained constant. The association vignette/background was randomized.

For humans, a session was composed of 1300 trials. All trial types were equiprobable (i.e. 100 trials per congruency level in each condition). In total, the group of human subjects performed approximately the same number of trials as each monkey.

### Evaluation of performance

Performance was recorded in terms of accuracy and response speed. We also computed minimal RT: we first calculated cumulative hit rate and false alarm rate across time for each subject using bins of 10ms; the minimal RT corresponds to the center of the first bin where the difference between false alarms and hits rates is significant (χ^2^ test, df = 1, α = 0.05).We performed statistical analyses using Matlab 7.6.0 (R2008a). The total number of trials across all human subjects was comparable with the number of trials from each monkey (15 human subjects, 1300 trials each, 19500 trials in total). We thus analysed data of each monkey separately, but pooled data of all human subjects. Before pooling, we applied a vincentisation procedure (see Results for more details) to normalise reaction times across subjects. Without this normalization, the fastest human subjects would have contributed disproportionately to the early part of the cumulated d’ calculation and to the latency computation.

We compared conditions within each monkey and within the pool of human subjects. To assess differences between conditions (congruent vs incongruent, and “noisy background” vs “real scene”) we calculated cumulated d-prime across time in each condition and performed permutation tests to determine whether the observed difference was significant. Statistical and mathematical calculations are detailed in the results.

## Results

### Global performance

Subjects, both monkeys and humans, performed the categorization task significantly above chance (Monkey Dy: 75.9% correct, Monkey Rx: 87.9% and humans: 78.4%, see Table 1 for more details). Moreover, as previously shown in other studies (Fize et al, 2011 [26]) both monkeys and humans were able to recognize animals regardless of the nature of the background (real scene, either natural or man-made, or noisy background). They made their decision based on the vignette identity and ignored the background.

**Table 1:** Global performance of subjects in the two conditions.

	Accuracy				Reaction times					
	Noisy Backgrounds		Real Scenes		Noisy Backgrounds			Real Scenes		
	Target	Distractors	Targets	Distractors	Minimum RT	Median RT	Mean RT	Minimum RT	Median RT	Mean RT
Monkey Dy	72%	71%	70%	88%	260ms	373ms	393ms	280ms	403ms	418ms
Monkey Rx	85%	93%	70%	93%	270ms	392ms	410ms	290ms	427ms	440ms
Humans	75%	83%	68%	87%	350ms	528ms	535ms	370ms	544ms	548ms

### “Real scene” Condition

In this condition, a vignette appeared on a real scene background. In congruent trials either an animal was pasted on a natural scene or an object on a man-made scene. In incongruent trials an animal was associated with a man-made scene and an object with a natural scene. Behavioral performance is summarized in Table 2.

**Table 2:** Go-response rate (hits and false alarms) as well as sensitivity (d’) in the “real scene” condition.

	Congruent			Incongruent			Congruency effect	
	Hit rate	False alarm rate	d’	Hit rate	False alarm rate	d’	Difference	p-value
Monkey Dy	77.0%	23.8%	1.45	66.2%	34.4%	0.82	0.63	<0.001
Monkey Rx	72.2%	4.4%	2.29	68.6%	8.5%	1.86	0.43	<0.001
Humans	70.6%	11.3%	1.75	66.4%	14.3%	1.49	0.26	<0.01

Both monkeys and humans had a higher hit rate (animals correctly categorized) and a lower false alarm rate (objects incorrectly categorized) in congruent trials than in incongruent trials.

We then calculated d’ (a measure of the signal detection theory) to quantify the congruency effect between the vignette and the background across response time (absolute time for both monkeys and relative time scale for humans, obtained after vincentisation).

#### Vincentisation

We used a vincentisation procedure to normalize reaction time distributions across subjects before pooling their data because individual variations were not negligible (median reaction times going from 478ms for the fastest subject to 584ms for the slowest, and standard deviation going from 65ms to 97ms). This method has the advantage of keeping the overall shape of the reaction time distribution while avoiding an overpowering influence of any individual participant.

We divided each subject’s global reaction time distribution into 20 classes (or quantiles) of equal duration, regardless of condition, trial or response type (i.e. respectively: “Real scene” or “Noisy background” condition, congruency level of trial, hit or false alarm). We then assigned a quantile number to each trial of each subject. Finally we pooled data of all subjects within the 20 quantiles.

#### Cumulated d’

The d’ is a sensitivity index which quantifies the ability to discern a meaningful stimulus (signal) from others (noise). It is calculated using the following formula: d’ = z(hit rate)-z(false alarm rate) where z is the inverse of the cumulative normal distribution.

The temporal evolution of d’ after stimulus onset can inform us about the time course of congruency effects. To calculate cumulated d’ as a function of response time (that is, the d’ based on all responses given before a particular time), we first calculated cumulated hit rates and false alarm rates as a function of response time, using constant bins of actual reaction times in monkeys and quantiles of reaction time in humans. For instance, cumulated hit rate for the q^th^ bin (or quantile) in a given condition “x” was obtained by:

$$HitRate_{x}\left(q\right)=\frac{\sum_{bin=1}^{q}nbHits{\left(RT\in bin\right)}_{x}}{nb\;T\;\text{arg}\;ets_{x}}$$

**Fig 3:**
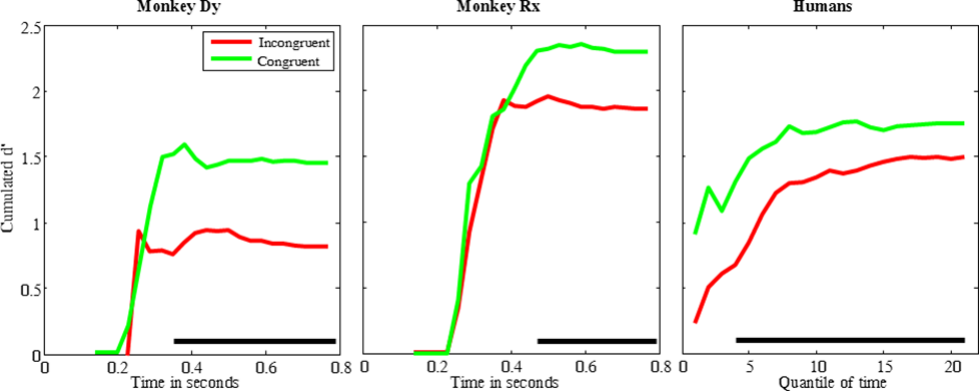
Cumulated D’ as a function of time in the “Real scene” condition (bins of 30ms in monkeys, quantiles of relative time in humans: mean quantile duration = 20ms SEM +/0.6ms, first quantile starts at 370ms SEM +/- 11ms). The horizontal black line indicates the significance of the difference between congruent and incongruent trials (permutation test, 1000 permutations, α<0.01)

**Fig 4:**
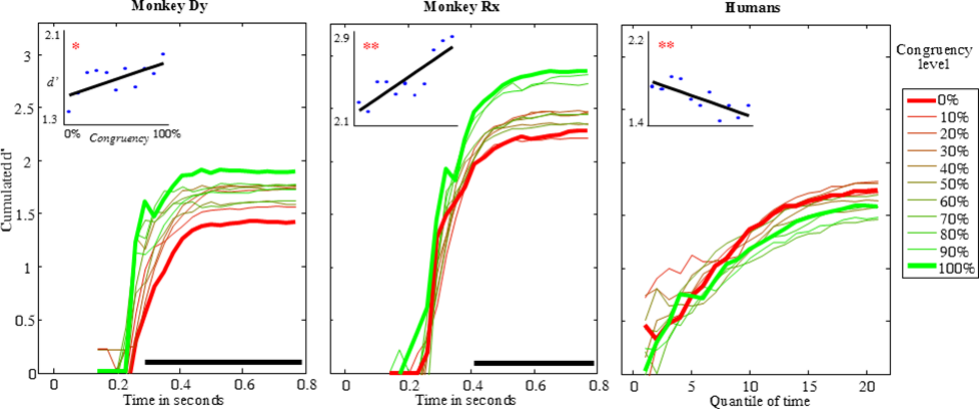
Cumulated d’ as a function of time in the “Noisy Background” condition (bins of 30ms in monkeys, quantiles of relative time in humans: mean quantile duration = 20ms SEM +/0.6ms, first quantile starts at 370ms SEM +/- 11ms). The thick red and green lines represent 0% and 100% congruency levels (respectively), while intermediate levels are depicted with thin lines. The horizontal black line indicates the significance of the difference between extreme congruency level trials (permutation test, 1000 permutations, α<0.01). Inserts on top left represent linear regressions of total cumulated d’ as a function of congruency level. Blue dots are observed values and black lines are the linear regression. Slopes, R^2^ and p-values are respectively s_Dy_ = 0.026, R^2^_Dy_ = 0.45 p_Dy_ = 0.024, s_Rx_ = 0.054, R^2^_Rx_ = 0.72, p_Rx_ = 0.001, s_H_ = -0.027, R^2^_H_ = 0.58, p_H_ = 0.006.

We finally applied the d’ formula to each bin or quantile and plotted the cumulated d’ curves as shown in Figs 3 and 4.

#### Permutation test

Permutation tests are a non-parametric approach to establishing the null distribution (expected distribution under the null hypothesis) of an experimental observation [32]. In our study, this observation is the value of the d-prime difference between the two conditions, congruent vs. incongruent. To establish the null distribution, we performed 1000 random permutations of the assignment of congruence labels (congruent/incongruent) to each trial. For each of the 1000 randomized data sets, we computed cumulated d’ for congruent and incongruent conditions and calculated the difference between the two conditions, exactly as done with the original experimental dataset. We thus obtained a distribution of theoretical differences under the null hypothesis at each reaction time bin (for monkeys) or quantile (for humans), and compared our observed difference values to this distribution. The ranking of the observed value among the theoretical distribution provides the p-value. If our observed difference value was higher than the last percentile of the null distribution, we considered it as statistically significant.

Both humans and monkeys discriminated animals from objects better on congruent backgrounds than on incongruent ones (Fig 3). This d’ difference reached statistical significance quite early (350ms after image onset for monkey Dy, 470ms for monkey Rx, and from the fourth quantile of time, i.e. 445ms SEM +/- 14ms in humans; permutation test, p<0.01), and remained significant until the end of the response window (limited to 800ms).

In short, humans and monkeys showed a similar contextual congruency influence in the categorization task with “Real scene” backgrounds.

### “Noisy Background” condition

In this condition, each background was constructed from a mixture of Fourier amplitude spectra from 100 images with a variable proportion of natural scenes and man-made scenes, and a randomized phase spectrum. Thus, the backgrounds looked like meaningless noise, but retained certain global statistical features of natural or man-made scenes (see Fig 2). Background/vignette congruency was gradually manipulated from 0% (incongruent) to 100% (congruent) by steps of 10%. In Table 3, results for the extreme conditions (0% and 100% i.e. incongruent and congruent) are summarized.

**Table 3:** Go-response rate (hits and false alarms) as well as sensitivity (d’) in the “Noisy background” condition.

	100% Congruent			0% Congruent			Congruency effect	
	Hit rate	False alarm rate	d’	Hit rate	False alarm rate	d’	Difference	p-value
**Monkey Dy**	73.8%	10.5%	1.89	64.3%	14.7%	1.41	0.48	<0.001
**Monkey Rx**	87.2%	4.3%	2.85	84.5%	10.1%	2.29	0.56	<0.001
**Humans**	73.7%	16.0%	1.58	77.2%	17.1%	1.74	-0.15	NS

While the results of both monkeys follow the same trend as in the real scene condition (higher hit rate and lower false alarm rate, higher d’ in congruent trials than in incongruent trials), it is not the case for human subjects, since their hit rate is higher and their d’ lower in incongruent trials.

When directly comparing d’ across extreme congruency levels, we found a significant difference (p<0.001) in both monkeys, in the same direction as in the real scene condition, i.e. d’ was significantly higher in congruent trials than in incongruent trials. However, in humans, there was no significant difference between d’ obtained for the two extreme congruency levels.

Finally, we computed the cumulated d’ with the same method as the one used in the “real scene” condition. This was done not only for the two extreme congruency levels (0% and 100%), but also for the 9 other intermediate levels. Results are shown in Fig 4.

Fig 4 shows the establishment of the congruency effect across time. In monkeys, the difference between extreme conditions (illustrated by horizontal black lines in Fig 4) appeared about 60ms earlier than in the real scene condition (from 290ms after stimulus onset for monkey Dy, and 410ms for monkey Rx) and remained stable across time. Moreover, the d’ values of the 9 intermediate congruency levels mostly fell between the 2 extreme levels. We computed linear regressions of total d-prime as a function of congruency level. For both monkeys, there was a significant positive correlation between congruency level and d’.

In humans, no significant difference between extreme congruency conditions was visible at any time point. From the tenth quantile onwards, d’ in the incongruent condition was actually slightly higher than in the congruent condition. Computing linear regression, we observed a significant negative influence of the congruency level on subjects’ performance (d’).

### Difference between conditions

To statistically evaluate the difference between congruency effects observed in the two conditions (real vs. noisy backgrounds), we performed random permutations of the assignment of trial condition. For each randomized data set we computed global d’, we calculated d’ difference (congruent vs. incongruent) within trial conditions, and finally we obtained a value of difference between conditions. We obtained a distribution of theoretical differences according to the null hypothesis (i.e. the two trial conditions do not lead to different congruency effects) and compared our observed value to this distribution. The null hypothesis could not be rejected for either monkey (Monkey Dy: observed difference = 0.15, p-value = 0.23, Monkey Rx observed difference = -0.13, p-value = 0.28, 1000 permutations); in other words, the congruency effect was not different in the 2 trial conditions. In humans, on the contrary, the congruency effect was significantly different in the two conditions (observed difference = 0.42 p-value = 0.013).

Altogether, these results imply that humans exhibited a contextual congruency effect only in the condition where the background was a real scene photograph; they did not process noisy backgrounds as real contexts. Monkeys, on the other hand, showed a similar contextual congruency effect than humans for real backgrounds, but this effect remained unchanged with noisy backgrounds.

## Discussion

The main goal of this study was to determine whether or not the amplitude spectrum of a scene could support, at least in part, the contextual congruency effect found in several categorization studies, in humans and monkeys. To answer this question we designed an ultra-rapid categorization task using two distinct trial types: either the context was a real scene photograph or a noisy background built from the average of 100 scenes’ amplitude spectra and random phase.

In the “Real Scene” condition, both humans and monkeys exhibited a similar congruency effect: they performed significantly better in categorizing vignettes when they were pasted on a congruent context than when they were pasted on an incongruent one. This result confirms those previously obtained by Fize et al, 2011 [26] with a similar task, and our observed congruency effect also has a similar magnitude. But, interestingly in our experiment we left aside the coherence of the object location within the scene (for instance a cow could appear in the sky of a landscape) and the object scale coherence (a bee could be as big as a rock) because vignette localization and context/vignette associations were randomized, and vignette sizes were equalized. In previous studies these two parameters were controlled. The collage of the vignette on the background was made in advance by the experimenter in order to create coherent stimuli in terms of scale and location, whereas in our experiment, it was performed automatically and randomly by the stimulation program. Our experiment thus indicates that the congruency effect exists at a superordinate category level, and is not or sparsely affected by spatial and scale coherence.

However, because in our task we did not counterbalance the target category, we cannot generalize this congruency effect obtained with animals as targets and man-made objects as distractors to the opposite categorization task, i.e. objects as targets and animals as distractors. This caution in generalizing our conclusions is all the more warranted since some studies indicate that living things are sometimes treated as a special visual category [33].

In the “Noisy Background” condition the backgrounds had amplitude spectra comparable to those of natural or man-made scenes (or various levels in-between). Because these amplitude spectra were obtained by averaging over 100 scenes from the same superordinate category (natural vs. man-made) but from various basic-level categories (e.g. street and indoor, sea and mountain, etc.), we can assume that the amplitude component was properly isolated from other potential physical features, including spatial layout which could be typical of scenes at the basic level [27].

Surprisingly we obtained different results in humans and monkeys: monkeys exhibited a similar congruency effect in the Noisy background condition as in the Real Scene condition while humans seemed to process noisy backgrounds in a different way than real scenes.

Monkeys better discriminated an animal from an object when the noisy background was built from amplitude spectra average of real scenes from the congruent category (man-made scenes for objects and natural scenes for animals) than on backgrounds built from incongruent category pictures. Interestingly, d’ congruency effects in the “Noisy Background” condition were not significantly different from those obtained in the “Real Scene” condition. So it seems that the congruency effect observed in monkeys relies in large part on image amplitude spectrum at the superordinate level. Moreover, regressing monkeys’ performance on the proportion of naturalness in these noisy backgrounds suggested that this effect can be progressively modulated, i.e. the congruency effect was proportional to the ratio of congruent over incongruent amplitude spectrum information.

On the contrary, humans’ performance was affected in a different way in this second condition. First we found no significant d’ difference between extreme congruency levels, i.e. an absence of congruency effect, although the same observers had displayed significantly positive congruency effects for real scene backgrounds. Further a linear regression taking into account the intermediate congruency levels showed that noisy backgrounds could actually give rise to a negative congruency effect: subjects were better able to discriminate animals from objects when the noisy backgrounds contained more incongruent physical features. The congruency effect obtained in the “Real Scene” condition thus cannot be explained by the amplitude spectrum of the scenes. Since there was no semantic information in the background (i.e. nothing to recognize or categorize), we might suggest that subjects processed it as a texture. Consequently, the negative influence of congruency on performance could be due to a type of camouflage effect: it might be easier to distinguish a curvilinear shape (i.e. an animal) on a background containing a lot of straight lines (i.e. features of a man-made scene) and conversely. This phenomenon has indeed been examined in visual search studies [34,35]; they concluded that it takes longer to find a target on a complex background whose features are similar to those of the target. In our experiment, presentation time was constant and very brief (50ms) and response time was limited (800ms), so this predicted decreased performance for background-congruent trials might be revealed as a lower d’ rather than an increased RT.

A congruency effect, positive or negative, implies three different mechanisms: object processing, scene processing and interactions between scene and object. Object processing has been extensively explored in both humans and monkeys [16, 36]; since objects were not manipulated in our experimental design, our results do not challenge those previously obtained for object categorization. To our knowledge, scene processing has been mainly studied in humans, in terms of category relevant features [27–30]. Moreover, background-object interactions and congruency effects have rarely been investigated in terms of physical features of the scene stimuli. Our study brought to light that there might be a difference between humans and monkeys in background processing, object-background interaction or both.

We can hypothesize that the observed difference between humans and monkeys may not be due to a difference in visual processing in itself, but rather to different visual experiences of the world. Congruency effects in humans would rely on associations between the to-be-categorized item and contextual elements which usually co-occur in the real world [17, 20, 37]. Since our monkeys were born and have always lived in captivity, they have very little experience of such real world co-occurrences; their visual system may thus need to analyze photographs of scenes as a collection of physical features. This notion raises the question of what representation monkeys may have of the scenes in our experiment. Many studies aimed to explore understanding of pictures by non-human primates. There are three possible ways of reading a picture, as defined in Fagot et al, 2000 [38]: The first one is the *independence* mode: the monkey does not link a picture to the real object; the second one is the *confusion* mode: the monkey confuses the picture with the real object; and the third is the *equivalence* mode: the monkey recognizes a picture as a representation of the real object. To determine which mode is used by monkeys, Truppa et al in 2009 [39] designed a Matching to Sample protocol with capuchin monkeys, and observed that these New World monkeys were able to match a real object with its photograph using the equivalence mode. In 2008, Parron et al. [40] tested this ability in pictorially naïve baboons, gorillas and chimpanzees. They first trained the animals to grasp a piece of banana presented against a pebble, and then tested them using stimuli pairs (a real object and a photograph or two photographs). Animals never mistook a real piece of banana for its photograph, but when a photograph of banana was presented against a pebble, both baboons and gorillas grasped it and tried to eat it, which suggests that they used the *confusion* mode. Lastly Pokorny and de Waal proved in 2009 [41] that capuchin monkeys were able to recognize photographs of group mate faces in an *equivalence* mode. Fagot et al. 2010 [42] suggest that recognition of a photograph by a monkey is a dynamic learning process. A monkey frequently exposed to photographs of well-known objects can learn to distinguish them. But on the other hand, it is possible that repeated exposure to pictures leads monkeys to adopt the independence mode. This could be the case in our study because 1) our monkeys were exposed to photographs for years, and 2) they never faced in real life most of the animals or objects we showed. In that case, monkeys would just read pictures as a collection of features, and learn to categorize items based on certain features without associating any meaning to the picture itself.
